# Prioritizing investments in rapid response vaccine technologies for emerging infections: A portfolio decision analysis

**DOI:** 10.1371/journal.pone.0246235

**Published:** 2021-02-11

**Authors:** Dimitrios Gouglas, Kevin Marsh

**Affiliations:** 1 Coalition for Epidemic Preparedness Innovations, Oslo, Norway; 2 Patient-Centered Research, Evidera, London, United Kingdom; Shandong University of Science and Technology, CHINA

## Abstract

This study reports on the application of a Portfolio Decision Analysis (PDA) to support investment decisions of a non-profit funder of vaccine technology platform development for rapid response to emerging infections. A value framework was constructed via document reviews and stakeholder consultations. Probability of Success (PoS) data was obtained for 16 platform projects through expert assessments and stakeholder portfolio preferences via a Discrete Choice Experiment (DCE). The structure of preferences and the uncertainties in project PoS suggested a non-linear, stochastic value maximization problem. A simulation-optimization algorithm was employed, identifying optimal portfolios under different budget constraints. Stochastic dominance of the optimization solution was tested via mean-variance and mean-Gini statistics, and its robustness via rank probability analysis in a Monte Carlo simulation. Project PoS estimates were low and substantially overlapping. The DCE identified decreasing rates of return to investing in single platform types. Optimal portfolio solutions reflected this non-linearity of platform preferences along an efficiency frontier and diverged from a model simply ranking projects by PoS-to-Cost, despite significant revisions to project PoS estimates during the review process in relation to the conduct of the DCE. Large confidence intervals associated with optimization solutions suggested significant uncertainty in portfolio valuations. Mean-variance and Mean-Gini tests suggested optimal portfolios with higher expected values were also accompanied by higher risks of not achieving those values despite stochastic dominance of the optimal portfolio solution under the decision maker’s budget constraint. This portfolio was also the highest ranked portfolio in the simulation; though having only a 54% probability of being preferred to the second-ranked portfolio. The analysis illustrates how optimization modelling can help health R&D decision makers identify optimal portfolios in the face of significant decision uncertainty involving portfolio trade-offs. However, in light of such extreme uncertainty, further due diligence and ongoing updating of performance is needed on highly risky projects as well as data on decision makers’ portfolio risk attitude before PDA can conclude about optimal and robust solutions.

## 1. Introduction

The Coalition of Epidemic Preparedness Innovations (CEPI) was set up in 2016 to support the development of vaccines for Epidemic Infectious Disease (EID) threats, contributing to the world’s preparedness for unexpected EID outbreaks [1–3]. A key strategic objective of CEPI has been to establish platform technology capabilities that can accelerate development, manufacturing and clinical evaluation of vaccines in response to outbreaks of newly emerging infections [1,4]; its importance exemplified by the world’s vaccine development response to the COVID-19 pandemic. In 2017, CEPI launched a Call for Proposals (CfP) to select a portfolio of platform technologies that would enable achievement of this strategic objective through an initial total investment of approximately US$ 140 million [5]. It was anticipated that supporting a diverse range of vaccine platforms could improve response to epidemic outbreaks by facilitating the rapid development of a novel vaccine should a previously unknown pathogen emerge [6]. Six platform projects that had participated in this CfP are now developing COVID-19 vaccines, several of which are in advanced clinical trials.

Platform technologies can generally be viewed as standardized, reproducible processes to develop and manufacture vaccines, which have previously been established through the development of other vaccines. Rapid response platforms can, in principle, improve the efficiency and overall timeframe of vaccine development; allowing for the start of clinical phase 1 testing just months after the viral sequence of a given pathogen is identified [6–9].

The decision to invest in the development of rapid response platforms to aid the response to the emergence of previously unknown pathogens faces challenges. First, whether an investment will generate benefit is subject to significant uncertainty. The successful development of a platform is highly uncertain, facing obstacles associated with organizational know-how and capabilities, technical and regulatory hurdles, and sustained utilization [10]. These challenges compound the well documented challenges of vaccine development–long timelines, scientific risks and operational complexities [11–15]. Assuming a platform is successfully developed, the benefit that the platform will deliver is subject to other sources of uncertainty [16], including: not knowing if the platform will enable the development of a vaccine that will protect against an unexpectedly emerging pathogen; and not knowing what the value of that protection will be–i.e., how many people would be put at risk by the pathogen and what risk the pathogen would pose to them.

Given these challenges, a single standardized financial or health-economic value metric is unlikely going to be able to measure the value of investments. In such a context, a multi-criteria value framework could be more appropriate, incorporating stakeholder preferences to inform how criteria should be traded off [17–19]. Such a framework would require the elicitation of preferences of relevant stakeholders involved.

Any such analysis is also likely to have to accommodate portfolio level effects. A single platform approach may be insufficient for rapid vaccine development in response to outbreaks caused by a multitude of unknown pathogens. Instead, a mix of platforms may be required to increase the likelihood of protection [6,20,21].

This study reports on a Portfolio Decision Analysis (PDA) application [22] to address the above challenges, and support CEPI’s investment decisions. To the best of the authors’ knowledge, no previous PDA to support pharmaceutical R&D has attempted to simultaneously test all above challenges–uncertainty in project evaluation; portfolio-level effects; and formally incorporating stakeholder preferences.

PDA has been increasingly used in R&D project selection across multiple application domains [22–26] due to its support in reducing the number of portfolio alternatives considered to a manageable size [27,28]; enhancing transparency through the consideration of all relevant criteria [22,24,25]; making relevant conflicts explicit [17,22,24]; accounting for the interconnectedness of projects [29] and providing insight about the overall value, cost and balance of a portfolio [29–32].

The increased use of PDA has also been seen in the field of pharmaceutical R&D decision making specifically [29–33]. This literature includes studies that address uncertainty and that incorporate stakeholder preferences. But to the best of the authors’ knowledge, no study has addressed both challenges simultaneously.

A commonly acceptable measure of uncertainty for pharmaceutical R&D portfolio decision-making is lacking [34]. Uncertainty has been addressed through use of decision tree analyses [33,35–37] as well as stochastic optimization methodologies [e.g. 34,38–47]. In all these studies the notion of uncertainty is partly conflated with that of risk of project failure, or inversely, probability of success (PoS). However, several studies introduce measures of uncertainty that capture variance of R&D portfolio performance more broadly, such as: Value at Risk (VaR) or Conditional Value at Risk (CVaR) [34], fuzzy value [43], reward/loss ratios [38,44,46] or value probability thresholds [34,38,40,44–46,48,49], variance of portfolio value distribution [39,42,44,50,51], semivariance below or above portfolio value thresholds [42], or covariance of portfolio value, cumulative probability distribution of portfolio value and Gini criteria [41]. A final set of methods emerging from the health economic literature attempt to measure the impact of this variance on the probability that a portfolio is chosen [42,52,53]. The main logic of these approaches is to generate model outputs in multiple iterations within a Monte Carlo simulation, and to determine, across all iterations, the proportion of outputs that fall favourably in relation to a given decision maker satisfaction threshold; allowing this way for probabilistic rankings to be constructed.

A handful of studies have formally incorporated decision maker preferences into PDA for pharmaceutical R&D [16,54,55], and few other studies have illustrated how preferences could be applied in hypothetical pharmaceutical R&D portfolio selection problems [43,44,46,48]. [54] employed an Analytic Hierarchy Process to assess the intensity of importance of decision criteria and alternatives in pairwise comparisons, allowing them to generate weighted scores to rank alternatives and to inform strategic investment decisions in a pharmaceutical company setting. [17] elicited stakeholder preferences using a swing-weighting technique, and then incorporated these into a multi-criteria decision analysis (MCDA) to evaluate projects, and consequently to generate an efficiency frontier. Building on the [17] model, [55] illustrated how optimal solutions along such a value-to-cost frontier can be generated when considering budget constraints and project interdependencies. [43] used fuzzy set theory to model imprecise and preference information associated with R&D project performance, project interactions, and stakeholder satisfaction degrees in resource constraint distributions, enabling the estimation of an optimal portfolio that maximizes monetary benefits under fuzzy resource constraints. A handful of other studies assumed stakeholder preferences as priority indices determining the sequencing of projects entering illustrative pharmaceutical R&D pipeline optimization problems [44,46,48]. However, no formal preference elicitation process, or outcome, was reported in any of these studies.

This study attempts to explicitly address uncertainty and formally incorporate stakeholder preferences into the optimization process. It does so through discrete choice modelling and testing of multiple uncertainty analysis methods within a stochastic optimization framework, in a real-life application with a high impact portfolio decision to be made. A commercially available simulation-optimization algorithm is employed to identify optimal portfolio solutions, and different uncertainty analysis techniques are compared to assess whether the identified solutions are also stochastically nondominated and robust.

## 2. Materials & methods

### 2.1.Scope and objective

The analysis focused on 16 platform projects that were submitted for an extended review following on the launch of a Call for Proposals (CfP) [5]. Projects were reviewed by CEPI between March and May 2018. The 16 platform projects had a combined budget of US$ 390 million, with budgets ranging from US$ 6 million to US$ 65 million, and with a median cost of US$ 22 million (see S1 Data). The goal of the PDA framework was to identify an optimal portfolio of platform technology investments that would maximize portfolio value under a US$ 140m budget constraint.

All projects were at the preclinical development phase, with the aim that CEPI funding would advance them through their testing against up to three pilot pathogens to the end of clinical phase 1. Projects covered 5 different types of platform technologies: RNA, Viral Vector, DNA, Protein, and gene-encoded mAb. Due to confidentiality restrictions, project owner/ development partner names have been anonymized throughout the remainder of this manuscript. Projects have been labelled as P1 to P16 and their grouping by platform type 1–5 is summarized in Table 1.

**Table 1:** Platform projects evaluated under the call for proposals for platform technologies to enable rapid vaccine development for epidemic prone infections.

Platform Type	Platform Projects
RNA (Platform Type 1)	P2 (mRNA), P7 (saRNA), P11 (mRNA), P16 (mRNA)
Viral Vector (Platform Type 2)	P4 (Replication-defective Chimpanzee adenovirus), P10 (Plasmid-Launched-Live-Attenuated Virus YF), P12 (Simian Adenovirus), P13 (Recombinant attenuated vesicular stomatitis virus)
DNA (Platform Type 3)	P3 (DNA-Needle Free Injection System), P8 (DNA-Electropolation Device), P15 (Lentiviral gene transfer vector)
Protein (Platform Type 4)	P1 (Nanoparticle-Subunit), P9 (Tobacco Mosaic virus—Virus Like Particle), P14 (Molecular Clamp Sub-unit)
Gene-encoded mAb (Platform Type 5)	P5 (Adeno Associated Virus-mediated monoclonal antibody), P6 (RNA vectored monoclonal antibody)

### 2.2.Study design

Six steps were undertaken to determine the optimal platform technology portfolio solution. First, project-level and portfolio-level evaluation factors were identified that were of interest to the decision makers, via stakeholder consultations and review of the literature. Second, these factors were structured into a platform technology portfolio valuation framework, accounting for parameter uncertainty. Third, expert assessments of platform project performance were collected and combined into performance estimates using a Monte Carlo simulation. Fourth, decision maker preferences were elicited on different types of technology platforms via a Discrete Choice Experiment (DCE). Fifth, project performance and decision maker preferences were combined in a simulation-optimization model to determine optimal portfolio solutions. Sixth, stochastic dominance and robustness of the optimization output were tested through a variety of uncertainty analysis techniques.

### 2.3.Step 1. Identifying evaluation factors

Stakeholder consultations (see S1 Appendix for details) identified the factors relevant to the evaluation of platform project portfolios. First, the probability of at least one project per platform type considered induces a sustainable, protection enabling accelerated vaccine R&D response to unexpected epidemic infection emergencies (PoS_≥1_). The consultation identified seven factors influencing PoS_≥1_ (see Table 2). Second, stakeholders suggested different value to PoS_≥1_ generated by different platform types and a non-linearity in preferences for PoS_≥1_.

**Table 2:** Factors influencing PoS of rapid response vaccine platform technology development projects.

Project PoS factor	Metric
C1. Applicant competency	Likelihood that the applicant is sufficiently competent to deliver on the proposed activities of the project
C2. Project feasibility	Likelihood that the project plans and procedures in place are of sufficient quality to ensure that three target pathogens are effectively investigated through to preclinical proof of concept, whereof two target pathogens are further effectively investigated through clinical Phase I studies
C3. Clinical benefit	Likelihood that the platform will enable immune responses providing protection/ clinical benefit against novel emerging infectious diseases on the basis of evidence provided on any pathogen
C4. Safety potential	Likelihood that the platform will be able to generate vaccines, with an acceptable safety profile, against novel emerging infectious diseases on the basis of evidence provided against any pathogens on the same platform
C5. Manufacturing scalability & speed	Likelihood that the platform will enable fast development and production, from design through clinical release of vaccine, in volumes sufficient to respond to outbreaks of novel emerging infectious diseases on the basis of evidence provided against each of the target pathogens and/or any other evidence provided on other pathogens as part of this application
C6. Operational suitability	Likelihood that the platform will enable stable storage and uncomplicated delivery of vaccine product in an outbreak response under extreme conditions
C7. Operational sustainability	Likelihood that the candidate platform developed through this project will remain in use and available to respond to newly emerging or unexpected pathogen outbreaks

### 2.4.Step 2. Defining R&D portfolio value

Based on the factors emerging from the previous step as relevant to the assessment of projects and of project portfolios, project PoS is defined per Eq (1) as the product of those factors contributing to the overall PoS of project *i* for a given technology platform type *k*. *N* indicates the total number of project PoS factors considered (which in this case is seven; for descriptions see Table 1). These factors were defined to be consequentially independent–i.e. the occurrence of one factor would not affect the probability of occurrence of others–even if some of these could potentially be correlated with each other in practice. This allowed for their multiplicative combination to generate overall project PoS estimates. The independence of the factors was ensured through the engagement of experts in the definition and structuring of the PoS factors. For instance, the risk of project failure because of staff competence (the inverse of factor C1) was deemed independent to the risk of project failure because of technical factors (C2-C5), as was the prospect that a project demonstrates clinical benefit (C3) but is not safe (C4), and vice versa.

$${\tilde{p}}_{i_{k}}=\prod_{N}{\tilde{C}}_{N_{i_{k}}}$$ (1)

Where:

${\tilde{p}}_{i_{k}}$ = platform project PoS

${\tilde{C}}_{N_{i_{k}}}$ = factors contributing to the overall PoS of platform project i

For each platform type *k*, the probability of at least one project being successfully developed is defined per Eq (2). This is calculated as the difference between 1 and the product of no project being successfully developed. *s* indicates here the total number of projects representing a technology platform type *k*. Here, the level of at least one successful project *POS*_*≥1(k)*_ associated with a given platform type *k* suggests that: a) more than 1 platform projects are being considered, at least one of which will succeed with a given probability; b) the PoS of each of these projects, as defined by Eq 1, will affect the overall probability of *POS*_*≥1(k)*_ for the platform type *k* they comprise.

$${\tilde{POS}}_{\geq 1(k)}=1-(\prod_{i_{k}}^{s}\left(1-{\tilde{p}}_{i_{k}}\right))$$ (2)

Where:

${\tilde{POS}}_{\geq 1(k)}$ = probability of at least one project being successfully developed for platform type k

As per Eq (3), overall portfolio value is defined as the weighted sum of products of *POS*_*≥1(k)*_ per platform type *k*. A weighting factor (${\tilde{w}}_{k}$) was added to the value function to reflect stakeholder feedback that their goal was more than simply maximising *POS*_*≥1(k)*_ and that the value of *POS*_*≥1(k)*_ varied between pathogens. It was not possible to define the source of this value more precisely and thus captured this as another factor in the value framework. Thus, the variation in the value of *POS*_*≥1(k)*_ by platform type was incorporated into the framework as a weighting factor and captured by eliciting stakeholder preferences (see methods step 4).

$${\tilde{V}}_{p}=\sum_{k=1}^{t}{\tilde{w}}_{k}∙{\tilde{POS}}_{\geq 1(k)}$$ (3)

Where:

${\tilde{V}}_{p}$ = Overall portfolio value

${\tilde{w}}_{k}$ = preference coefficient for platform type k

*t* = total number of platform types k included in the portfolio

#### 2.4.1.Step 3. Generating project PoS estimates (C1, C2, C3, C4, C5, C6, C7).

Each project *i* was quantitatively assessed against PoS factors *C1*_*i*_ to *C7*_*i*_ by four to five reviewers, each of whom assessed three to four projects, ensuring their balanced assignment in terms of numbers as well as representation of required review competencies per project (see S1 Appendix for details). Overall, a total pool of 27 reviewers was used for assessment of projects.

For each of *C1*_*i*_ - *C7*_*i*_, reviewers were asked to define the most likely, worst-case and best-case outcomes for each project. Reviewers provided initial assessments online (step 3.1 –initial reviewer assessments) and final assessments following a face-to-face meeting (step 3.2—final reviewer assessments). Results of project assessments against *C1*_*i*_ - *C7*_*i*_ were combined to estimate projects’ overall PoS as per Eq (2), through a random sampling process (10,000 iterations). In each iteration a reviewer was randomly selected and a PoS factor estimate was randomly drawn from that reviewer’s distribution, assuming the reviewers’ estimates defined a triangular distribution, and factors were combined as described in Eq (1). Across iterations of the simulation it was then possible to estimate the mean and variance in projects’ PoS.

#### 2.5.Step 4. Eliciting platform preferences (w_k_)

A DCE [56] was employed to help elicit stakeholder preferences for platform types, denoted as *w*_*k*_. A DCE elicits from survey participants’ their choices between pairs of decision options [57]. The options are described using a pre-defined set of attributes, such that the analysis of choices can be used to generate a utility function which describes how variation in attributes contributes to the preference for an option [57].

DCE participants involved 48 individuals, comprising a diverse group of expert stakeholders: 27 external expert reviewers; 8 CEPI expert staff; and 13 members of the Scientific Advisory Committee (SAC). The SAC is CEPI’s formal governance body responsible for making recommendations for funding to the CEPI Board. It is an independent, expert and invested group, under no obligation to agree with expert reviewer assessments or with formal investment decisions made by the CEPI Board. At the time of this CfP, the SAC comprised: 8 representatives of governments, regulators and multilateral organizations; 7 representatives of non-profit R&D organizations; 6 academics; 4 industry representatives; and 4 independent subject-matter experts [58].

The 48 DCE participants were given a series of choice sets, in which they were asked to choose between portfolio alternatives defined by different levels of achievement (*POS*_*≥1(k)*_). For each platform type (the attributes), this likelihood was defined as one of three levels of achievement (Table 3). Given lack of published evidence on rapid platform project PoS, and given the time constraints on the analysis, the levels included in the choice model were informed by the initial reviewer assessments (step 3.1).

**Table 3:** Attributes and levels of achievement employed in the DCE.

Platform types (*k*)	Lower level (*a*)	Middle level (*b*)	Upper level (*c*)
RNA (Platform Technology Type 1)	(*POS*_*≥1*_ *= 0%)*	(*POS*_*≥1*_ *= 30%)*	(*POS*_*≥1*_ *= 60%)*
Viral Vector (Platform Technology Type 2)	(*POS*_*≥1*_ *= 0%)*	(*POS*_*≥1*_ *= 30%)*	(*POS*_*≥1*_ *= 60%)*
DNA (Platform Technology Type 3)	(*POS*_*≥1*_ *= 0%)*	(*POS*_*≥1*_ *= 28%)*	(*POS*_*≥1*_ *= 56%)*
Protein (Platform Technology Type 4)	(*POS*_*≥1*_ *= 0%)*	(*POS*_*≥1*_ *= 20%)*	(*POS*_*≥1*_ *= 40%)*
Gene-encoded mAb (Platform Technology Type 5)	(*POS*_*≥1*_ *= 0%)*	(*POS*_*≥1*_ *= 6%)*	(*POS*_*≥1*_ *= 12%)*

*POS*_*≥1(k*)_ represents the likelihood of successfully developing at least one project by platform type *k*.

**Fig 1:**
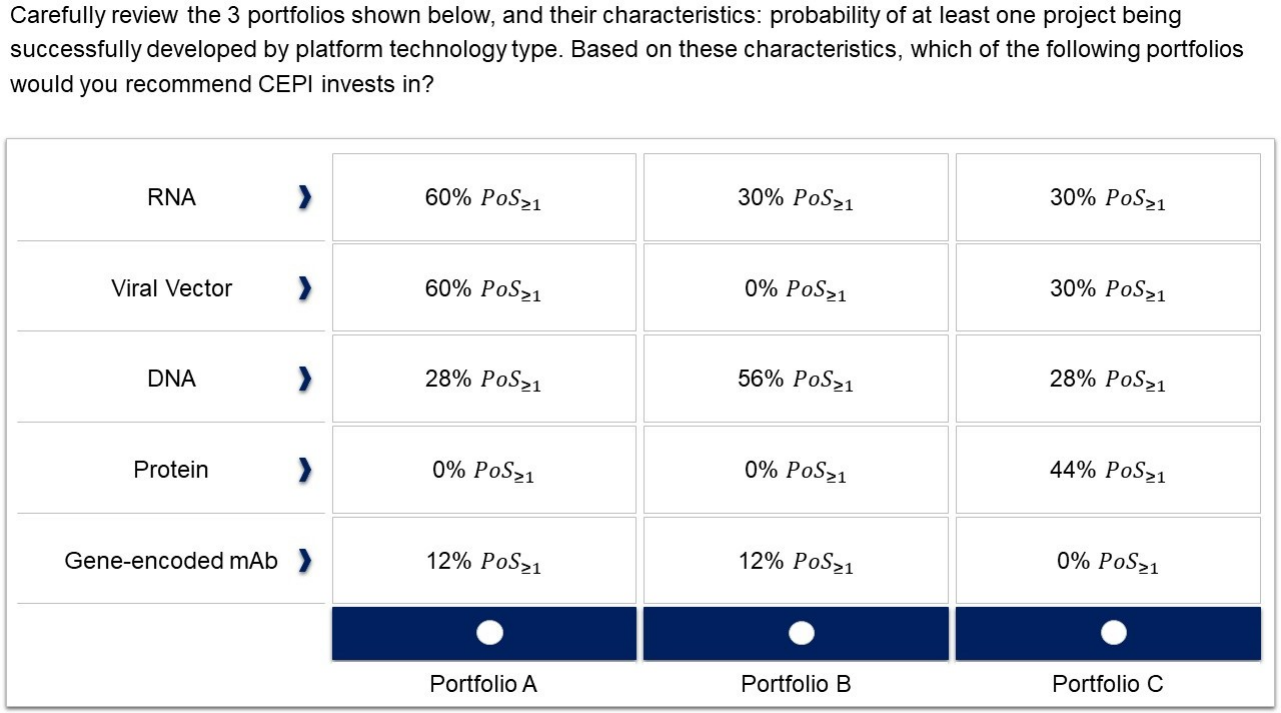
Example choice set in the DCE.

Each choice set comprised three portfolios (see example in Fig 1). An experimental design of 2 blocks of 16 choice sets (32 choice sets in total) was generated using JMP® Pro 13.2.1 software. In order to minimize bias in responses, the order of the attributes within each choice set and of the choice sets within each survey was randomized between DCE participants, and the experimental design was assessed for orthogonality and balance. Internal validity of responses was assessed through dominance and consistency tests (see S1 Appendix for details).

Participants’ choices were analysed using a conditional logistic regression of the following form and applied using JMP®, Version 13. SAS Institute Inc., Cary, NC, 1989–2007:

$$U_{j}=\sum_{k=1}^{t}\left[\beta_{b(k)}X_{b(k)}+\beta_{c(k)}X_{c(k)}\right]$$ (4)

Where:

*U*_*j*_ = *Utility produced by portfolio choice j*

*X*_*b*(*k*)_ = *the middle level* (*b*) *of PoS*_≥1_ *performance for platform type k*

*X*_*c*(*k*)_ = *the upper level* (*c*) *of PoS*_≥1_ *performance for platfrom type k*

*β*_*b*(*k*)_ = *Part worth associated with moving from the lower level of PoS_≥1_ performance (a) to the medium level of performance (b) on platform type k*

*β*_*c*(*k*)_ = *Part worth associated with moving from the medium level of PoS_≥1_ performance (b) to the upper level of performance (c) on platform type k*

Results of the model were used to estimate preference functions *w*_*k*_ for the different platform types as per Eq (5), where values *w*_*k*_ are estimated depending on whether *POS*_*≥1(k)*_ falls within a *b—a* versus a *c—b* range of achievement in the choice model (see Table 2); *a* being the lower level of *POS*_*≥1(k)*_ achievement (which is equal to zero), *b* the middle level of *POS*_*≥1(k)*_ achievement and *c* the upper level of *POS*_*≥1(k)*_ achievement, for each platform type *k* considered in the choice model.

$${\tilde{w}}_{k}=\left\{ \begin{array}{c} {\tilde{\beta }}_{b(k)}\cdot \frac{1}{b-a},if\;{\tilde{POS}}_{\geq 1(k)}\leq b\\ \frac{{\tilde{\beta }}_{b(k)}}{{\tilde{POS}}_{\geq 1(k)}}+\frac{{\tilde{\beta }}_{c(k)}}{{\tilde{POS}}_{\geq 1(k)}}\cdot \frac{{\tilde{POS}}_{\geq 1(k)}-b}{c-b},if\;{\tilde{POS}}_{\geq 1(k)}>b \end{array}\right.$$ (5)

*Where*:

${\tilde{\beta }}_{b(k)}$ = *stochastic parameter of β_b(k)_, following a Normal distribution*

${\tilde{\beta }}_{c(k)}$ = *stochastic parameter of β*_*c*(*k*)_*, following a Normal distribution*

Given the anticipated heterogeneity in stakeholder preferences, *w*_*k*_ was modelled as a stochastic preference parameter in the overall value function described in Eq (3), drawing randomly (10,000 iterations) from the respective platform type’s utility coefficient distribution fit to the DCE data. Utility coefficients generated from conditional logistic regression models are normally distributed, justifying the distributional choices in Eq 5.

### 2.6.Step 5. Constructing optimal portfolios

To construct optimal portfolios a mathematical programming problem was solved using a simulation-optimization algorithm provided by the Analytic Solver® platform of FrontlineSolvers®. The R&D portfolio selection problem was to select a set of platform projects from a pool of candidate projects that maximizes portfolio value under a given budget constraint. Since performance uncertainty and preference heterogeneity were expected to be encountered in making R&D project portfolio decisions, a stochastic mixed integer programming model was designed to support optimal R&D portfolio decisions in an uncertain R&D environment, per Eq (6).

$$\underset{x}{\arg\;\max}\;f(x)≔\{x|f(x)={\tilde{V}}_{p}=\sum_{k=1}^{t}{\tilde{w}}_{k}[1-(\prod_{i_{k}}^{s}\left(1-{\tilde{p}}_{i_{k}}X_{i_{k}}\right))]\}$$ (6)

$$s.t.\;\sum_{\begin{array}{c} 1\leq i_{k}\leq s\\ 1\leq k\leq t \end{array}}B_{i_{k}}X_{i_{k}}\leq B$$ (6.1)

$$X_{i_{k}}\{0,1\}\forall i_{k}$$ (6.2)

**Table:** 

***Indices and sets***		***Parameters***	
*i*∈*I* *k*∈*K* *V*_*p*_ *B* *s* *t*	Projects Technology platform types Value of the portfolio Budget available The total number of projects representing a technology platform type The total number of technology platform types	${\tilde{p}}_{i_{k}}$ ${\tilde{w}}_{k}$ $B_{i_{k}}$	PoS distribution of project *i* representing technology platform type *k* Preference coefficient for a given ${\tilde{POS}}_{\geq 1}$ in a technology platform type *k*. Budgetary cost of project *i* representing technology platform type *k*
		***Variables***	
		$X_{i_{k}}\left\{ \begin{array}{c} 1,\;if\;project\;i\;representing\;platform\;type\;k\;is\;selected\\ 0,\;\mathrm{otherwise} \end{array}\right.$	

Given the above non-smooth optimization problem formulation has many potentially feasible solutions, the Analytic Solver® platform’s evolutionary algorithm was used to identify optimal portfolios. The algorithm starts by randomly drawing from a population of candidate solutions. It learns and adapts its search for better optima in relation to a current solution, as the composition of the population of candidate solutions changes. This adaptation is supported by random changes to the original population of candidate solutions, yielding new and improved candidate solutions. Throughout this process, an evolutionary algorithm selects the fittest and eliminates the least fit candidate solutions.

Given that the optimization objective function depends on multiple, stochastically independent uncertainties, the evolutionary algorithm applied to the objective of maximizing expected *V*_*p*_ is unlikely to identify the highest *V*_*p*_. Instead, *V*_*p*_ was maximized given chance constraints, defined as the percentile of the values computed for this objective function, across trials of the Monte Carlo simulation. Specifically, the addition of a chance constraint *VaR*_*a*_ *V*_*p*_ *≥ 95%* to the optimization model allowed the identification of portfolio solutions with the highest *V*_*p*_ under different budget constraints, which other model runs did not when maximizing by expected *V*_*p*_ or by *V*_*p*_ against *50% ≤ VaR*_*a*_ *V*_*p*_ *≥ 90%*. Varying project allocations under different budget constraints from US$ 6 million (lowest budget of the evaluated projects) to US$ 390 million (total budget if all projects were to be considered), the model was also able to identify optimal portfolio solutions along an efficiency frontier.

### 2.7.Step 6. Uncertainty analysis

To further test the impact of uncertainty on the optimization, all possible portfolio alternatives were first identified under the US$ 140 million constraint, through multiple optimization runs (approximately 40,000 runs), each time marginally varying the budget constraint (by approximately US$ 0.0003 million). For each portfolio alternative under the given budget constraint, their mean, variance, semivariance, absolute deviation, and the mean-Gini statistic were then estimated, allowing for stochastic dominance testing (see S1 Appendix for details).

A probabilistic sensitivity analysis was also conducted to test robustness of the optimal portfolio solution, by estimating the rank probability of portfolio alternatives. This was done in pairwise comparisons between the optimal portfolio and all alternative portfolios identified at the US$ 140 million budget constraint. For each pairwise comparison, the pair of portfolios were ranked in each of 10,000 simulation iterations, and the probability that the optimal portfolio would outrank each of these portfolio alternatives by *V*_*p*_ was estimated across all iterations.

## 3. Results

### 3.1.Project PoS

**Fig 2:**
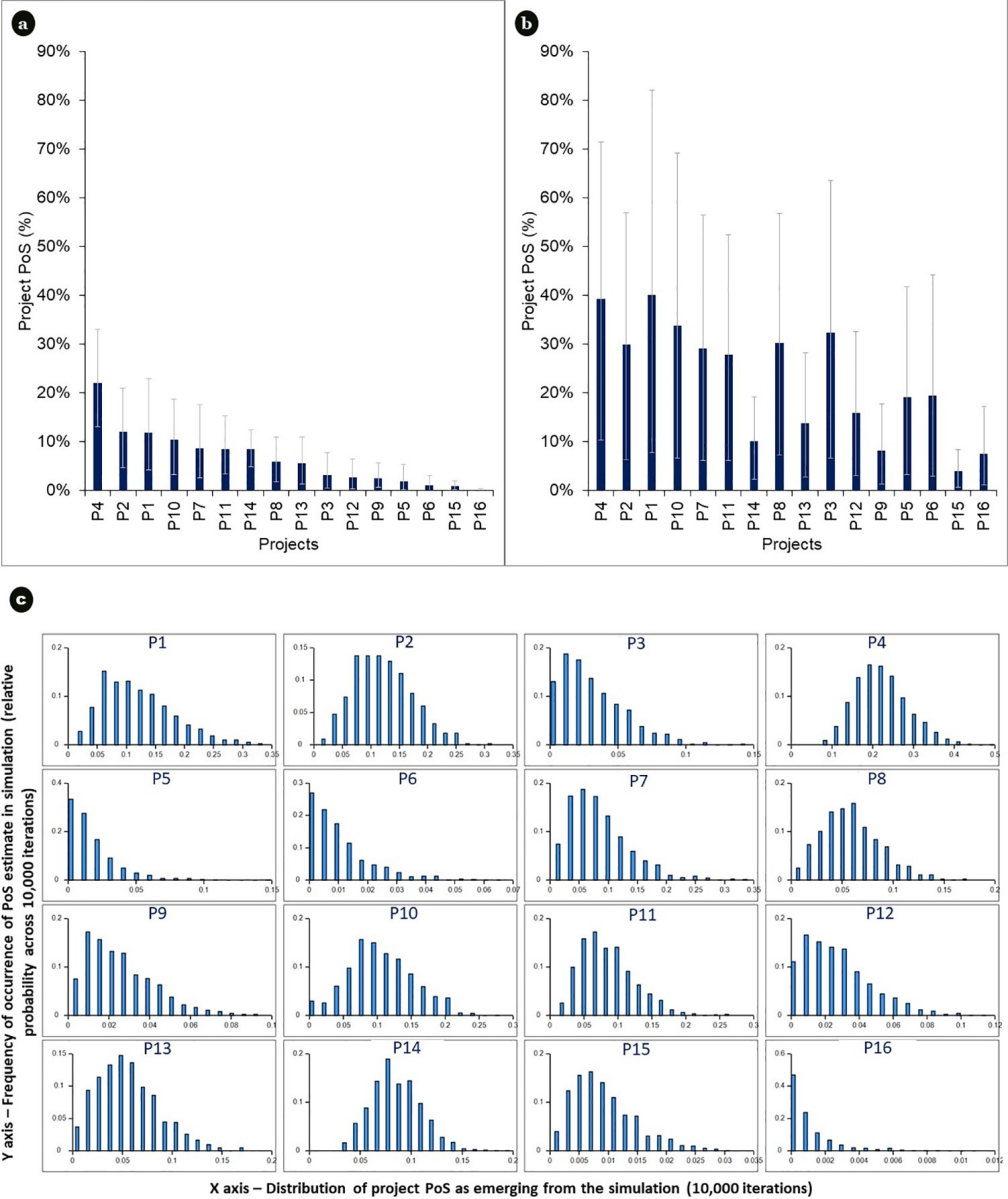
a, b. Project PoS (Mean, 95% CI). Displaying the mean and variance in PoS of projects generated by the simulation (10,000 iterations) under methods steps 3.1 and 3.2 (initial versus final reviewer assessments). c. Project PoS distributions (final reviewer assessments). Displaying the final project PoS distributions for the 16 projects assessed. Each bar chart represents another project, with the vertical axis indicating the frequency of occurrence of PoS estimates out of 10,000 simulation iterations, and the horizontal axis indicating different levels of PoS estimates emerging across the 10,000 simulation iterations.

Fig 2A–2C present the PoS distributions of the 16 projects, based on initial *versus* final reviewer assessments. They demonstrate that PoS estimates substantially overlap between projects, though final PoS estimates are significantly lower than per initial assessments, changing the rank ordering of projects by PoS, within and between platform types.

### 3.2.Portfolio preferences (w_k_)

Table 4 shows the choice model estimated from the DCE. It demonstrates how the utility that stakeholders place on a chosen portfolio varies with the probability of at least one project successfully developed per platform type *POS*_*≥1(k)*_. Stakeholders attach different value to *POS*_*≥1*_ generated by different platforms. For instance, there is a non-overlap between confidence intervals in 0–30% *POS*_*≥1(k)*_ gains of RNA *versus* Protein and in 0–28% gains of Viral Vectors *versus* DNA. Moreover, there are consistently decreasing returns to investing in increasing *POS*_*≥1(k)*_ of a single platform type. For instance, stakeholders prefer a gain of 0% to 30% in *POS*_*≥1(k)*_ of RNA to the same gain in *POS*_*≥1(k)*_ of other platforms. However, once it goes above 30%, the incremental return on *POS*_*≥1(k)*_ for RNA becomes less, justifying diversifying the portfolio into other platform types.

**Table 4:** Choice model derived from responses to the DCE survey.

Term	Utility Coefficient (*β*)	Std Error	Lower 95%	Upper 95%	p value
RNA (Platform Type 1)_ *POS*_*≥1*_ [0%-30%]	1.313	0.081	1.156	1.474	<0.001
RNA (Platform Type 1)_ *POS*_*≥1*_ [30%-60%]	0.360	0.070	0.223	0.498	<0.001
Viral Vector (Platform Type 2)_ *POS*_*≥1*_ [0%-30%]	1.167	0.082	1.009	1.329	<0.001
Viral Vector (Platform Type 2)_ *POS*_*≥1*_ [30%-60%]	0.463	0.070	0.326	0.600	<0.001
DNA (Platform Type 3)_ *POS*_*≥1*_ [0%-28%]	0.833	0.076	0.685	0.984	<0.001
DNA (Platform Type 3)_ *POS*_*≥1*_ [28%-56%]	0.118	0.073	-0.026	0.261	0.11
Protein (Platform Type 4)_ *POS*_*≥1*_ [0%-20%]	0.710	0.077	0.560	0.861	<0.001
Protein (Platform Type 4)_ *POS*_*≥1*_ [20%-40%]	0.266	0.075	0.121	0.413	<0.001
Gene-encoded mAb (Platform Type 5)_ *POS*_*≥1*_ [0%-6%]	0.133	0.073	-0.011	0.277	0.07
Gene-encoded mAb (Platform Type 5)_ *POS*_*≥1*_ [6%-12%]	-0.043	0.076	-0.193	0.106	0.57

AICc = 2706.86; BIC = 2760.6; -2*LogLikelihood = 2686.73.

**Fig 3:**
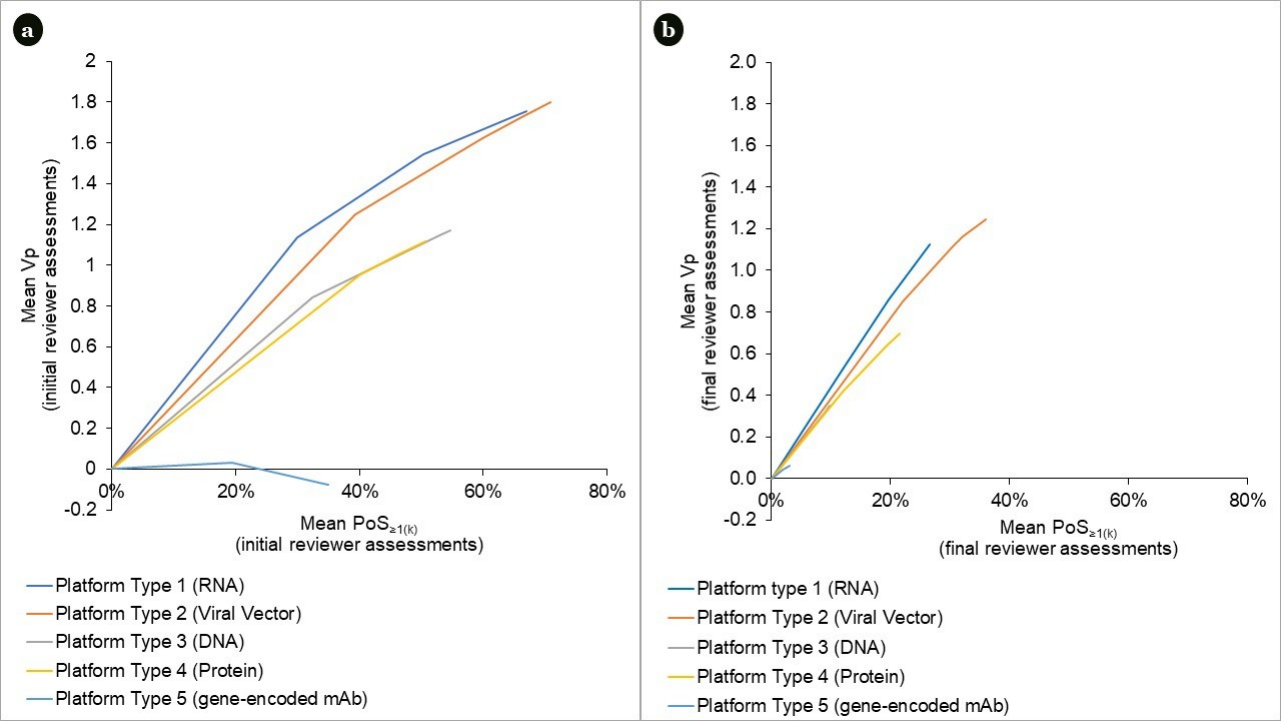
a. Portfolio value associated with probability of ≥1 project successfully developed per platform type (initial reviewer assessments). Mean POS_≥1(k)_ and V_p_ estimates are calculated by running the optimization process under step 5 separately for each platform type *k*, as follows: maximizing V_p_ several times, each time incrementally increasing the number of projects (decision variables in the model) entering the portfolio, and repeating this process until all projects are added. b. Portfolio value associated with probability of ≥1 project successfully developed per platform type (final reviewer assessments). Mean POS_≥1(k)_ and V_p_ estimates are calculated by running the optimization process under step 5 separately for each platform type *k*, as follows: maximizing V_p_ several times, each time incrementally increasing the number of projects (decision variables in the model) entering the portfolio, and repeating this process until all projects are added.

Fig 3A shows the cumulative value of projects, grouped by platform type and ordered by *POS*_*≥1*_ as identified through the initial reviewer assessments. Fig 3B shows the same output, but drawing from final reviewer assessments. As PoS estimates in this step were reduced, even cumulative PoS often were not as high as the mid-points in the preference function and thus failed to reflect the non-linearities in stakeholder preferences. This was an artefact of study timelines necessitating the design of the DCE based on initial reviewer assessments.

### 3.3.Optimal portfolios

**Fig 4:**
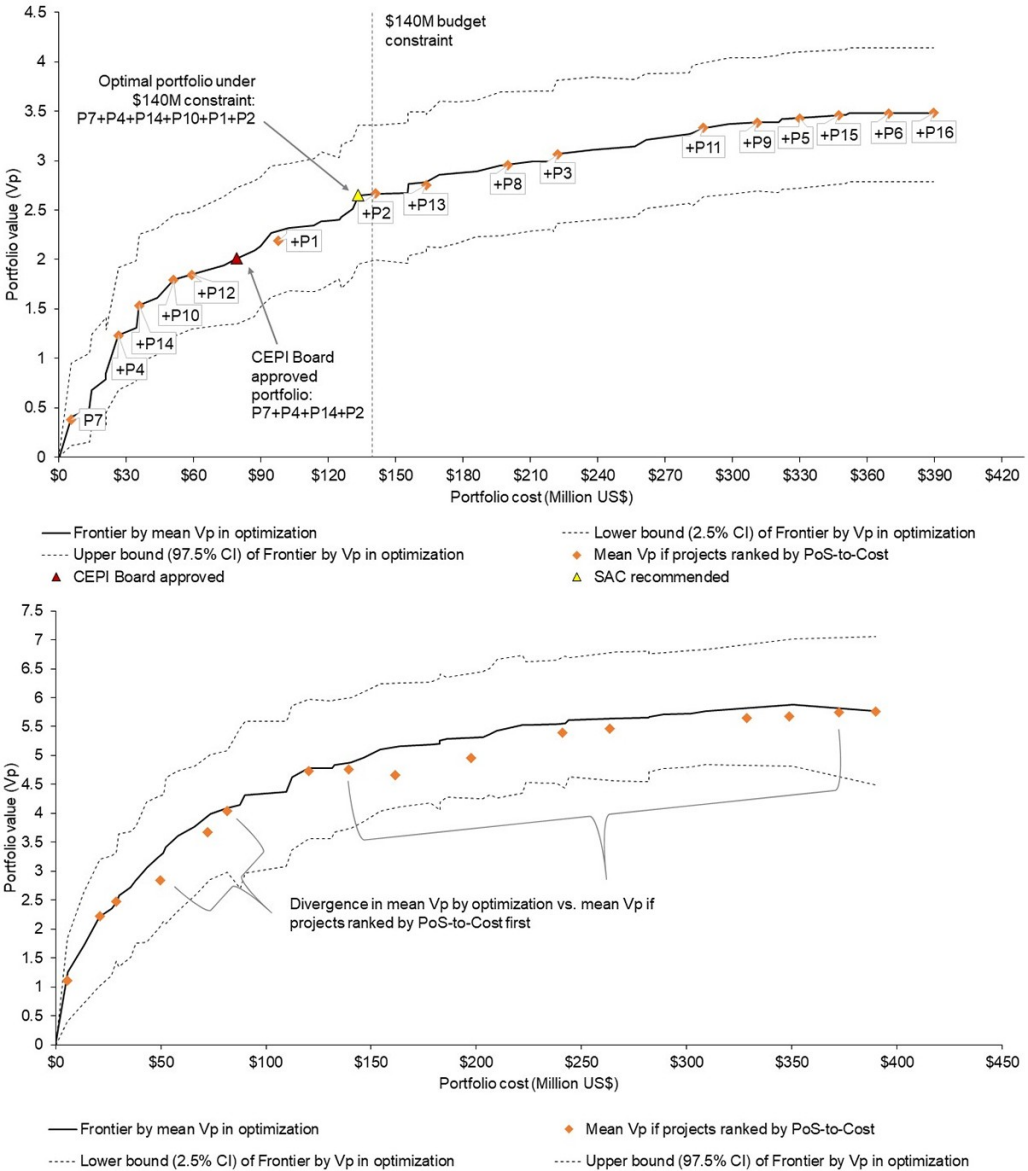
a. Optimal Frontier by maximizing portfolio value drawing from final reviewer assessments of project PoS. Fig 4A shows the efficiency frontier constructed by the optimization process under step 5, drawing from final reviewer assessments of project PoS. This is compared against the frontier that would have been generated if projects were simply ranked by expected PoS-to-Cost, then incrementally added to the portfolio without accounting for whether the resulting portfolios would maximize V_p_ under different budget constraints. b. Optimal Frontier by maximizing portfolio value drawing from initial reviewer assessments of project PoS. Fig 4B shows the efficiency frontier constructed by the optimization process under step 5, drawing from initial reviewer assessments of project PoS. This is compared against the frontier that would have been generated if projects were simply ranked by expected PoS-to-Cost, then incrementally added to the portfolio without accounting for whether the resulting portfolios would maximize V_p_ under different budget constraints.

Fig 4A demonstrates the optimal portfolio solution under the US$ 140 million budget constraint, which was also the SAC recommendation to CEPI–composed of the two best performing projects under each of the platform technology types 1 (RNA), 2 (Viral Vector), and 4 (Protein). The portfolio that was finally approved for funding by the CEPI Board excluded 1 Viral Vector and 1 Protein project from this recommended portfolio. This followed further due diligence of the recommended projects by internal CEPI expert teams. This portfolio was also positioned on the optimal value-to-budget frontier. Fig 4A also demonstrates which projects would have been selected if ranked by their PoS-to-Cost–including a third Viral Vector project (P12) that the Board did not approve but excluding one RNA project (P2) that was approved for funding.

Fig 4B shows the optimal frontier updated to draw from the initial reviewer assessments of project PoS. In this case, the impact of the non-linear preference function becomes more evident, as reviewer assessments are distributed across the ranges of performance reflected in the levels in the DCE.

### 3.4.Uncertainty analysis

**Fig 5:**
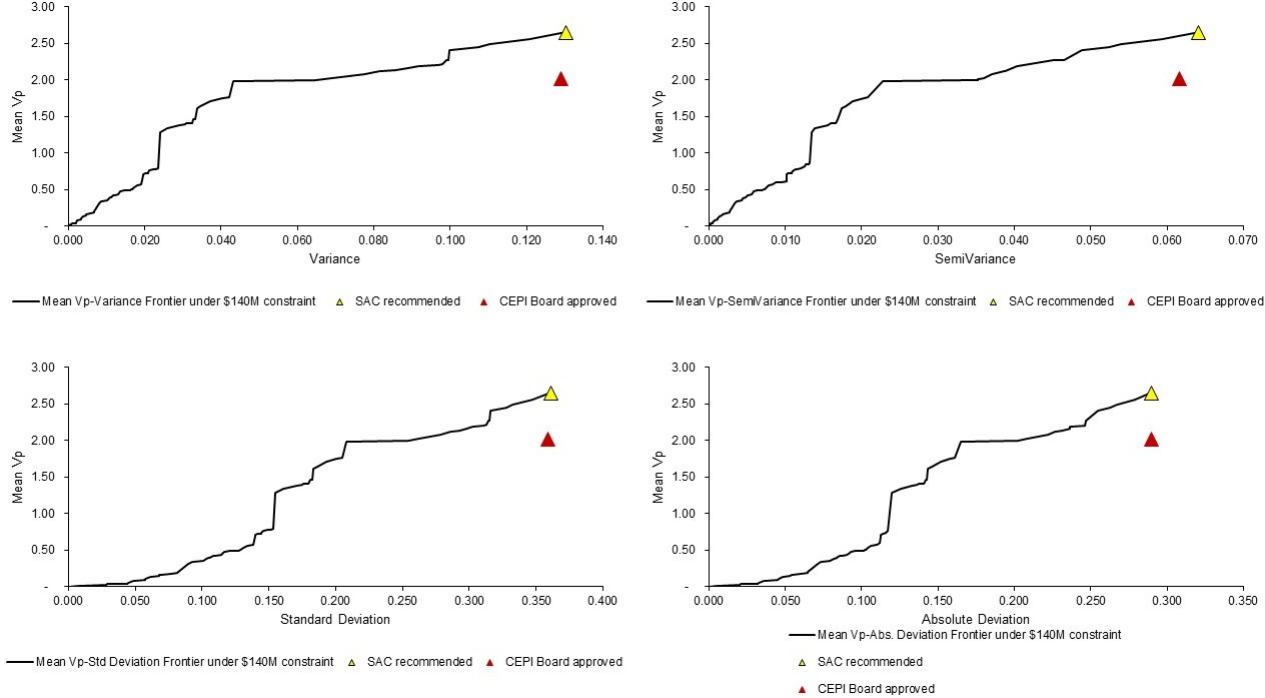
Optimal frontiers by mean-variance, mean-semivariance, mean-standard deviation, and mean-absolute deviation, under a US$140 million constraint.

The confidence intervals presented in Fig 4A demonstrate the large amount of uncertainty in final portfolio valuations. Fig 5 compares the optimal (and SAC recommended) portfolio and CEPI Board approved portfolios with alternatives under the decision maker’s budget constraint through various means of variance–mean-variance, mean-semivariance, mean-standard deviation, and mean-absolute deviation. This suggests that the optimal portfolio is stochastically dominant to the CEPI Board approved portfolio. In addition, no portfolio alternative with lower, equal or higher variance than the SAC recommended portfolio has an equal expected value.

The notion of stochastic dominance tested in Fig 5 requires assumptions about the shape of decision makers’ utility function and the shape of the probability distribution of the optimization outcomes, which appear to not be in line with the CEPI Board concerns about the level of acceptable risk present in the optimal portfolio. Assessment of stochastic dominance using the Mean-Gini relaxes these assumptions, just requiring that decision makers are risk averse [42,59].

**Fig 6:**
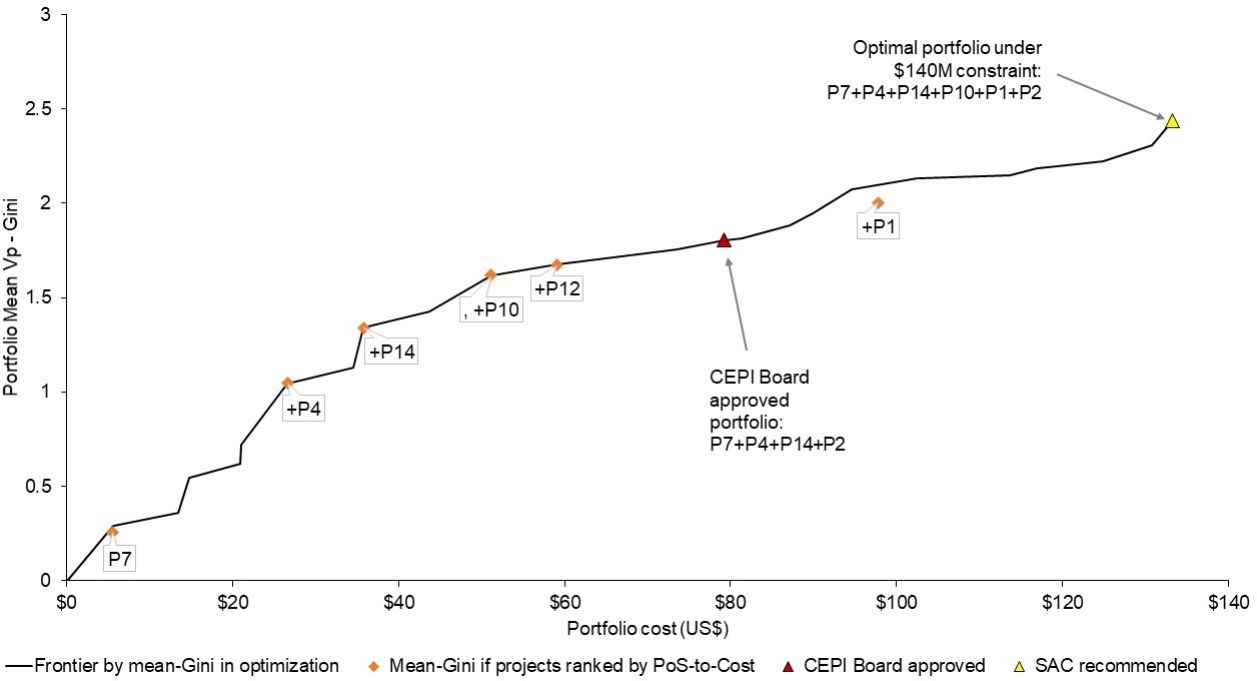
Optimal Frontier by Mean-Gini performance of the portfolio, under a US$140 million constraint.

Fig 6 illustrates the optimal value-to-budget frontier under a US$140 million constraint using the Mean-Gini statistic. Given that the optimal portfolio solution has both the highest mean *V*_*p*_ (Figs 4, and 5) and the highest mean-Gini statistic (Fig 6), this analysis confirms stochastic dominance of the optimization solution, given the assumptions on decision maker attitudes to risk underlying these models. Similarly to Fig 4A, the mean-Gini to budget analysis marginally differentiates from findings of a simple ranking of projects by PoS-to-Cost.

**Fig 7:**
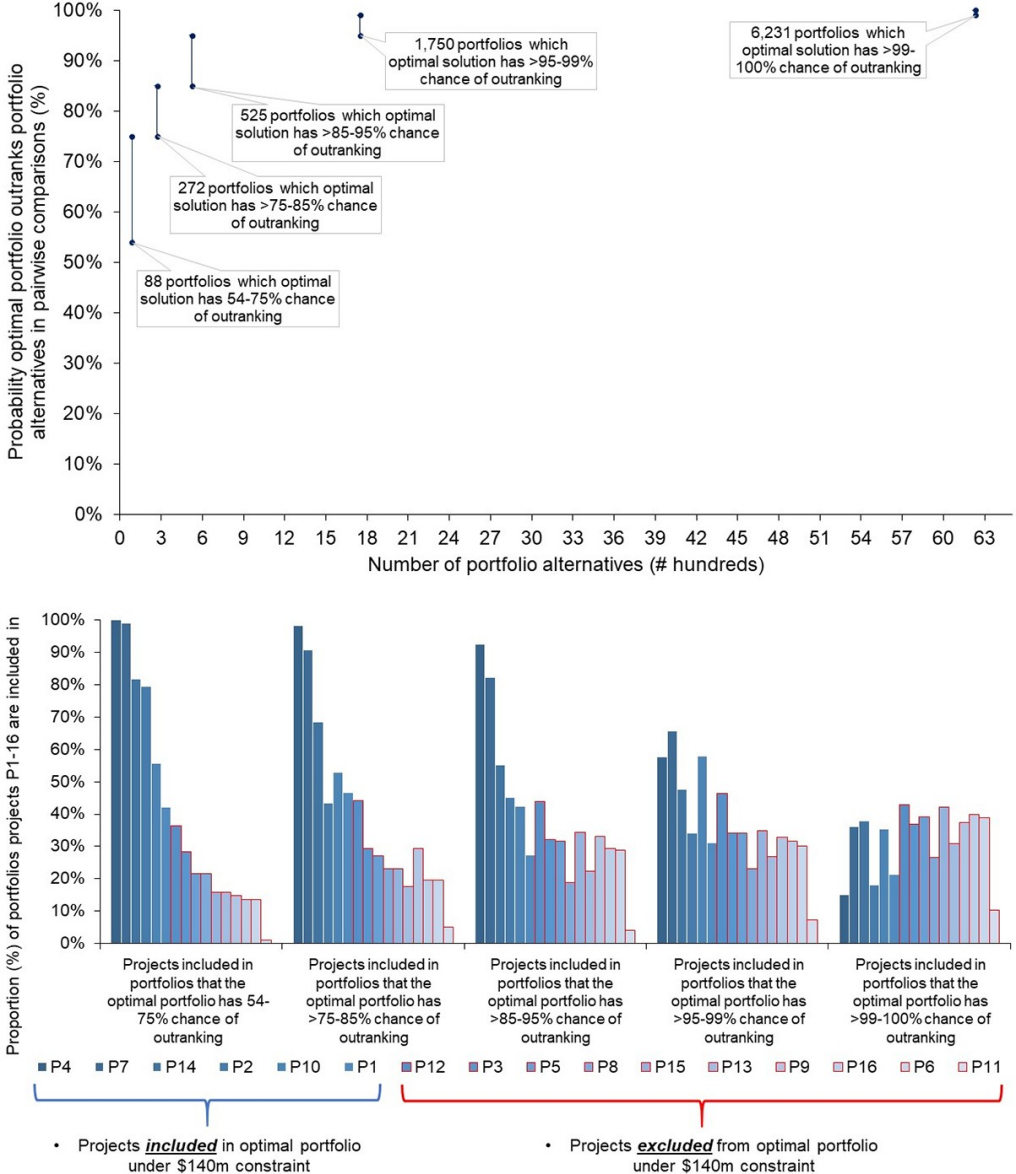
a. Probability ranges of optimal portfolio outranking alternative portfolios under a US$ 140 million constraint. b. Project composition of portfolio alternatives the optimal portfolio outranks under a US$ 140 million constraint.

Fig 7A illustrates that out of all pairwise comparisons between the optimal solution and each of the 8,866 unique alternatives with a budget under US$ 140 million, the optimal portfolio had a >54–100% chance of outranking portfolio alternatives. Fig 7B illustrates to what extent the chance of the optimal portfolio outranking other portfolios changes according to changes in their project composition.

The optimal portfolio under the budget constraint has a 54% probability of outranking the second-best portfolio by value. The latter comprises one less project under platform technology type 4 (Protein), one additional project under platform technology type 2 (Viral Vector), and one new project under platform technology type 3 (DNA).

Projects P4, P7, P14 and P2 are included in over 80% of the 88 portfolios that are outranked by the optimal portfolio by a 54–75% chance in the simulation. Projects P10 and P1, which were not approved for funding by the CEPI Board, are included in only 56% and 42% of these portfolios. The probability that other portfolios outrank the optimal portfolio decreases as the extent that projects P4, P7, P14 and P2 are excluded from these portfolios increases.

## 4. Discussion & conclusions

This study has reported on a PDA designed to support a global health R&D funding entity in making decisions to invest in platform technology projects to support response to unknown pathogen outbreaks. The funder faced significant uncertainty and portfolio selection trade-offs. This was particularly so in terms of the future use potential of platforms, but also in terms of the probability that platforms would be successfully developed and that they would be effective in the face of outbreaks.

There are three sets of findings that can be drawn from the study. First, the optimization output corresponded with the SAC’s recommendation to CEPI to fund 2 RNA, 2 Viral Vector and 2 Protein platform projects. However, the two riskiest of the six projects were eventually not approved for funding by the CEPI Board. This raised questions about the robustness of the PDA solution relative to decision makers’ attitude to portfolio risk.

The optimization demonstrated a positive correlation between the expected value of a portfolio and the variance around this estimate, suggesting a higher risk that the portfolio does not achieve the mean expected value. Despite this, various uncertainty analysis methods indicated that the optimal portfolio is also stochastically nondominated, restricted in their conclusions however by assumptions on decision makers’ attitudes to risk. The Monte Carlo Simulation suggested that this portfolio only had a 54% probability of ranking first compared with the second-best portfolio by value; and that this ranking probability was particularly sensitive to the downside risk of two out of the six projects comprising this portfolio. Whereas the sensitivity analysis was able to identify those downside risks, lack of information on decision makers’ attitude to portfolio risk prevented the PDA from concluding as to portfolio robustness during the CfP process. This would include data on how decision makers trade-off increasing expected value and increasing variance in expected value, and data on the acceptable level of outranking probability. Several studies illustrate how this could be done by setting limits on the variation around an R&D portfolio’s expected value (e.g. see [40,46,52]).

Practically, this finding also points to the importance of experience-based feedback to sequential updates of previous investment decisions as more information emerges about project strengths and risks. Mean-variance analyses ignore the impact of these learnings central to technology choice problems [60], which are dynamic in nature and require regular monitoring of progress of investments. The multi-armed bandit literature (e.g. [61,62] offers alternative perspectives on how portfolio choices can be made when decision-makers are faced with uncertainty. Here, the emphasis is on avoiding negative outcomes and particular attention is given on the dynamic process for decision making. The importance of such a sequential strategy for managing uncertainty has been illustrated elsewhere in different ways [63,64]: with uncertainty in health product development gradually diminishing as candidates advance through development phases, more information about their actual potential is revealed, and periodic updates of portfolio decisions at key stage gates ensure returns are optimized. Regardless of whether one uses a multi-criteria decision analysis framework, statistical decision indices [65], real options [35] or other decision tree approaches [33], the common need in such a process is adaptation of models to new knowledge about portfolio performance and to evolving decision-maker priorities.

Second, the study illustrates some of the methodological challenges, and potential solutions, facing PDA in the context of early health R&D investment decisions. The analysis demonstrated that uncertainty was particularly evident in the likelihood that investments could generate platform projects that would be effective in face of an unexpected epidemic infection emergency. This is reflected in reviewers’ assessments of project PoS. Given the uncertainties of whether any technology platform project can ever be applicable as well as rapidly respond to multiple such disease epidemics [8,66], it is unsurprising that project PoS estimates were low, despite the optimism of initial assessments informing the model; and with substantial variation.

The PDA considered the impact of this uncertainty and stakeholder preferences for different platform types on portfolio assessment; albeit preferences’ impact on portfolio valuation was smaller than anticipated, given how levels in the choice model were set–drawing directly from the initial reviewer assessments, in absence of published benchmarks on rapid platform PoS. Because of this, capturing the diminishing returns to investing in the same platform types using the DCE generated only a marginally different optimality frontier than just focusing on project PoS-to-cost rankings.

Portfolio preferences have been captured in other studies by the introduction of a diversity criterion or constraint, for instance by: structuring R&D portfolios by disease area, platform technology type, or early *versus* late phase of development of projects considered (e.g. see [29,32,46,50,67–69]), imposing a limit on the allocation of resources between project types by strategic goal (e.g. see [43]), or restricting resource allocation between R&D activities because of resource dependencies (e.g. see [44,48]). In practice, platform potential emerges through the accumulation of evidence of performance against a variety of diseases. This point is exemplified by the experience with the accelerated development of several CEPI funded vaccines using RNA, Viral Vector, Protein and DNA platforms in response to the COVID-19 pandemic [70]. However, all projects considered in this study were at the same early stages of development. Moreover, there are extreme uncertainties around their use potential against multitudes of unknown pathogens if successfully developed in off-epidemic conditions. Valuing the portfolio by disease area or different phases of development would therefore be a challenging task. Other limit setting approaches would be less relevant as allocation limits are already a function of the *POS*_*≥1(k)*_ and of constraints specified in the model.

This study modelled the optimization problem as a nonlinear stochastic problem and used an evolutionary algorithm to solve it. A key limitation of the evolutionary algorithm is premature convergence, i.e. the loss of diversity between sets of solutions too quickly in the solution search process, which can lead to outcomes that are not globally optimal. To avoid this, an optimization problem can be transformed to a linear or smooth problem, reducing its complexity and addressing the challenge of non-convergence. Ultimately, however, there is a degree of choice in how one models real-life problems and a trade-off that one needs to make between accurate reflection of real-life complexity and model simplification for computational efficiency and precision. In this study, the identification of all portfolio alternatives helped confirm the optimality of the solution generated by the model. This was possible because the optimization problem was small but would be an intractable exercise in larger problems, where model transformations would allow for globally optimal solutions to be found in more efficient ways.

Finally, several practical limitations with the elicitation of stakeholder portfolio preferences were identified. First, the time constraints facing decision makers are not always amenable to rigorous preference elicitation. Second, in the context of sample size limitations, as is often the case when working with expert groups, there are limitations on the complexity of the value models that can be characterized by choice models, such as DCEs. However, this will be less of a concern when stakeholders’ values of interest to decision makers is a larger group. In healthcare settings this typically relates to patients or the general population. In this study, it was the values of a broader set of experts beyond those (SAC members) making formal recommendations to the CEPI Board, which were of interest to decision-making. Consequently, the considerations of these values made it also practically possible for a DCE to be employed as the stakeholder group was large enough relevant to the number of attributes and levels considered in the model. In addition, logistical limitations meant that it was necessary to elicit preferences using a survey. This decision was vindicated by the results of the choice analysis, which was sufficiently precise to be able to differentiate the utilities associated with many of the levels in the choice sets. Other preference elicitation methods could also be employed, such as workshop-based swing weighting (e.g. [17]), however such methods are generally restricted by practical constraints of time, location and availability of stakeholders engaged.

The analysis demonstrates that while optimization modelling can help decision makers identify optimal portfolios in the face of significant decision uncertainty and portfolio trade-offs, in the presence of such problem characteristics further data on decision makers risk attitude is required before PDA can conclude about the optimal portfolio. Collecting such data will, however, face practical constraints. It will be necessary to identify such requirements early in the decision-making process, so that time and resources are available to elicit decision makers’ preferences in the context of health R&D decision making.

## Supporting information

S1 Appendix (DOCX)

S1 Data (XLSX)
