# Supplementary material for: Prioritizing investments in rapid response vaccine technologies for emerging infections: A portfolio decision analysis
Source: PLoS One. 2021 Feb 11;16(2):e0246235. doi: 10.1371/journal.pone.0246235 (PMC7877621; doi:10.1371/journal.pone.0246235)
Supplement: S1 Appendix — (DOCX) [file pone.0246235.s001.docx]

# **S1 Appendix**

*Article title*: Prioritizing investments in rapid response vaccine technologies for emerging infections: a portfolio decision analysis

*Journal name*: PLOS One

This document provides supplementary detail on: (1) the methodology used to identify evaluation factors; (2) the methodology used to define performance distributions for assessing platform project proposals; (3) the methodology used to design and test the optimality of the discrete choice experiment employed for elicitation of portfolio preferences in the optimization model; and (4) definitions of stochastic dominance using different uncertainty analysis tests.

## **Supplementary information on Methods Step 1: Identifying evaluation factors**

### **PoS factors**

A list of potential factors that informed the PoS of a project was generated from semi-structured interviews with 11 members of the SAC, 10 CEPI staff (internal experts), and a review of: previous CEPI evaluation frameworks [1]; the CEPI Business Plan [2]; evaluation criteria used by other supporters of platform technology development – such as the WHO [3], BARDA [4], and the US Department of Defense [5,6]; and published literature [7-12]. All SAC members and select CEPI expert staff (33 individuals in total) were consulted on the initial list to determine which factors were most relevant to CfP decisions.

To narrow down the list of PoS factors, and combine these into the analytical framework, members of the SAC and CEPI staff were engaged first in an email survey and then in one-to-one interviews between October 2017 and December 2017 to determine: whether all PoS factors relevant to CfP decisions had been captured; the relationship between the factors, and whether any of these factors should be removed or re-grouped if overlapping, or irrelevant. Based on this feedback, the research team developed a final list of seven factors contributing to project PoS, which was approved by CEPI’s Scientific Advisory Committee (SAC) in February 2018 (Table 1). PoS factors were defined in such a way to ensure stochastic independence – i.e. the occurrence of one factor would not affect the probability of occurrence of others – allowing for their multiplicative combination to generate overall project PoS estimates (see equation (2) in the main manuscript).

**Table 1**: **Factors influencing PoS of rapid response vaccine platform technology development projects.**

| **Project PoS factor** | **Metric** | **Considerations that influence performance against the PoS factor** |
| --- | --- | --- |
| C1. Applicant competency | Likelihood that the applicant is sufficiently competent to deliver on the proposed activities of the project | - Technical competency/expertise of project staff - Experience in preclinical testing of vaccines - Experience of the applicant in executing Phase I/II clinical vaccine trials - Experience of the applicant in regulatory interactions with relevant authorities and licensing of vaccines - The applicant’s vaccine manufacturing capabilities and skills |
| C2. Project feasibility | Likelihood that the project plans and procedures in place are of sufficient quality to ensure that three target pathogens are effectively investigated through to preclinical proof of concept, whereof two target pathogens are further effectively investigated through clinical Phase I studies | - The platform concept/ scientific rationale and ability to reach the 16-week timeline from antigen identification to product release for clinical trials - The development plan through to preclinical proof-of-concept for all three target pathogen vaccines - The early clinical development plan through to the end of Phase I for two target pathogen vaccines - The regulatory approach for advancing the project from preclinical through to Phase I - The process development and manufacturing plans, either in-house or via contract manufacturing partners |
| C3. Clinical benefit | Likelihood that the platform will enable immune responses providing protection/ clinical benefit against novel emerging infectious diseases on the basis of evidence provided on any pathogen | - Current evidence on the platform’s ability to induce robust immune responses in humans - Potential of the proposed immunological response testing approach to provide adequate characterisation of the proposed platform in Phase I trial - Potential of proposed platform to induce robust immune responses in humans against two target pathogens - Potential of proposed platform to induce robust immune responses in humans against novel emerging infectious diseases as demonstrated by evidence on any pathogens - Probability of meeting a 6-week timeframe from administration of first dose to achievement of immunologic protection/clinical benefit |
| C4. Safety potential | Likelihood that the platform will be able to generate vaccines, with an acceptable safety profile, against novel emerging infectious diseases on the basis of evidence provided against any pathogens on the same platform | - Quality of the current safety evidence on the proposed platform (including preclinical toxicology data, e.g. neurovirulence, and biodistribution studies, studies where relevant) - Potential of proposed platform to demonstrate an acceptable safety profile for the vaccines against the target pathogens, based on assumptions and evidence to date - Potential of proposed platform to demonstrate an acceptable safety profile for vaccines against novel emerging infectious diseases as demonstrated by the rationale and evidence of any pathogen on the same platform, based on safety record in humans (no of individuals exposed) including preliminary data in children and other potential risk groups |
| C5. Manufacturing scalability & speed | Likelihood that the platform will enable fast development and production, from design through clinical release of vaccine, in volumes sufficient to respond to outbreaks of novel emerging infectious diseases on the basis of evidence provided against each of the target pathogens and/or any other evidence provided on other pathogens as part of this application | - The process and release testing analytical methods and (including potency assays) to monitor process performance and expedite product release - Quality of the evidence and rationale on the platform’s manufacturing performance and yield (if previous experience with the proposed platform exists) - Potential of the proposed platform to support rapid manufacturing, formulation, fill and finish of a 10,000/ 100,000/500,000/1,000,000 dose equivalent of bulk and vaccine product for clinical testing and emergency use - The anticipated Cost of Goods in manufacturing of 10,000/ 100,000/500,000/1,000,000 dose equivalent of bulk and vaccine product for clinical testing in an emergency response |
| C6. Operational suitability | Likelihood that the platform will enable stable storage and uncomplicated delivery of vaccine product in an outbreak response under extreme conditions | - Proposed platform’s potential to deliver vaccines in minimal dosing schedules, in emergency situations - Proposed platform’s potential to support stability of vaccine product ensuring a shelf life ≥6 months at temperature suitable for long term storage and reactive use of the vaccine - Proposed platform’s feasibility for use in emergency response situations including potential requirement for delivery device |
| C7. Operational sustainability | Likelihood that the candidate platform developed through this project will remain in use and available to respond to newly emerging or unexpected pathogen outbreaks | - Evidence and rationale on platform’s routine ongoing use for other pathogen vaccines - Evidence and rationale on platform’s availability and use, via viable in-house or contract manufacturing partner facility operations, after the end of the project |

## **Supplementary information on Methods Step 3. Generating project PoS estimates (C1, C2, C3, C4, C5, C6, C7)**

Projects were quantitatively assessed against PoS factors C1 to C7 by 27 reviewers. Each project was assessed by four to five reviewers. They were selected through an open competitive process based on demonstrable experience – including years of work experience – in non-clinical, clinical, chemistry, manufacturing and control aspect of vaccine development. CEPI assigned reviewers to evaluate specific projects based on subject matter expertise on EID vaccine development and avoiding conflicts of interest. Reviewers received a manual and presentation providing detailed descriptions of PoS factors, scorecard templates, instructions and examples for filling in these templates. Further assistance and clarifications were provided in response to specific questions over email and phone, throughout the review process.

For each of C1-C7, reviewers were asked to define the most likely, worst-case and best-case outcomes for each project. Reviewers were first asked to provide individual assessments (between February and March 2018) and to submit these online using a customized reviews submission platform at CEPI. Reviewers then met in a face-to-face meeting (April 2018), and were asked to update their individual assessments, if needed, following group discussions on technical merits of the projects considered.

The results of the reviewer assessments of projects against C1-C7 were combined to estimate projects’ overall PoS through a random sampling process (10,000 iterations). In each iteration a reviewer was randomly selected and a PoS factor estimate was randomly drawn from that reviewer’s distribution, assuming the reviewers’ estimates defined a triangular distribution. Factors were combined as described in equation (1) provided in the main manuscript. The mean and variance in project PoS across the iterations was then estimated.

Inter-reviewer variability was assessed for worst-case, most-likely and best-base estimates for each of C1-C7 based on the average difference of individual reviewers’ estimates from the average estimate across all reviewers. Based on [13] classification, reviewer variability was assessed as good if it deviated less than 20% from the average, and excellent if it deviated less than 10% from the average. 7 of the 27 reviewers were found to have provided an estimate that deviated more than 20% from the average. In total, these deviations accounted for only 4% of the total number of worst-case, most likely, and best-case estimates collated from all reviewers, for all criteria (see S2 Data file for reviewer response data). These deviations reflected genuine differences of expert opinion, even after projects were thoroughly discussed during a face-to-face reviewer meeting.

## **Supplementary information on Methods Step 4. Eliciting platform preferences (**$\mathbf{w}_{\mathbf{k}}$**)**

At least three key statistical properties need to be met to ensure optimality of DCE designs [14]: D efficiency, orthogonality, and balance. First, the software generated 10,000 alternative designs in order for the most optimal design to be selected based on the D efficiency statistic [15-17] – a statistical measure commonly used to select the most efficient, though only fractional, factorial design, useful in situations where all combinations of the levels of the attributes are not possible to include. Given five attributes, each with three levels, considered in this DCE, a full factorial design would need to consider 243 choice sets, which would be practically prohibitive and tedious for DCE participants.

Second, statistical independence between attributes – which is a desired property of orthogonality in fractional factorial DCE designs [14] – was tested by computing pairwise correlations between attributes and their levels considered in the selected design. The highest correlation was 0.3, and the average correlation was 0.02, suggesting high orthogonality of the selected design. Third, manual edits to the DCE design were made to remove any dominant choice sets; and in doing so to improve the balance of the design, that is to ensure that each attribute level would occur equally often in the DCE, minimizing the variance in the parameter estimates.

Three other choice sets were added to each of the two blocks of choice sets: a practice question; a dominance test; and a consistency test. The inclusion of these tests intended to help clarify to what extent DCE respondents appropriately attended to the choice tasks. 75% of DCE survey respondents provided a consistent response and 85% correctly addressed the dominance question. When the probability that the dominance question was preferred was modelled based on the choice model [18] it was estimated that only 69% of respondents would be expected to select the dominant option, suggesting that DCE respondents attended to the task.

## **Supplementary information on Methods Step 6. Uncertainty analysis**

A solution would be deemed as stochastically dominant in two alternative ways. According to the mean-variance statistics, stochastic dominance would be achieved if: a) the solution’s expected value being greater than or equal to other portfolio alternatives for a given level of risk (equation (1.1)); and b) its variance being smaller than or equal to other portfolio alternatives for a given expected value (equation (1.2)):

$E\left( V_{p(a)} \right)\geq E\left( V_{p(b)} \right)$ *(1.1)*

*and*

$\sigma_{a}\leq\sigma_{b}$ *(1.2)*

*Where:*

$E\left( V_{p(x)} \right)$ *= expected value of portfolio x*

$\sigma_{x}$ *=variance of portfolio x*

According to the mean-Gini statistic, stochastic dominance of the solution would be achieved if: a) its expected value being greater than or equal to other portfolio alternatives (equation (2.1)); and b) the distance between the expected value and twice the covariance of expected value and cumulative probability distribution of portfolio value being greater than or equal to other portfolio alternatives (equation (2.2)) [19,20]. A version of the Gini statistic as used in this study is reported in equation (2.3), also previously employed in [19,20].

$E\left( V_{p(a)} \right)\geq E\left( V_{p(b)} \right)$ *(2.1)*

*And*

$E\left( V_{p(a)} \right) - \Gamma_{V_{p(a)}}\geq E\left( V_{p(b)} \right) - \Gamma_{V_{p(b)}}$ *(2.2)*

*Where:*

$\Gamma_{V_{p}} = 2cov\left[ V_{p}, F\left( V_{p} \right) \right]$ *(2.3)*

*Notations:*

$\Gamma_{V_{p}}$ *= Gini statistic*

$E\left( V_{p(x)} \right) - \Gamma_{V_{p(x)}}$*= Mean-Gini statistic for portfolio x*

$F\left( V_{p} \right)$ *= Cumulative Distribution Function of* $V_{p}$

# **S1 APPENDIX REFERENCES**

[1] Gouglas D, Marsh K. Prioritizing investments in new vaccines against epidemic infectious diseases: A Multi-Criteria Decision Analysis. JMCDA 2019;26(3-4):153-163. <https://doi.org/10.1002/mcda.1683>.

[2] CEPI. Coalition for Epidemic Preparedness Innovations preliminary business plan 2017–2021. 2016. [Cited 2019 16 December]. Available from: <http://cepi.net/sites/default/files/CEPI%20Preliminary%20Business%20Plan%20061216.pdf>.

[3] WHO. Research and Development Blueprint: Evaluation of ideas for potential platforms to support development and production of health technologies for priority infectious diseases with epidemic potential. 2016. [Cited 2019 16 December]. Available from: <https://www.who.int/medicines/ebola-treatment/R-D-Blueprint_Evaluation-of-platform-technologies-for-priority-patho.pdf?ua=1>.

[4] BARDA. The BARDA BAA Medical Countermeasures Acquisition Process. 2018. [Cited 2019 16 December]. Available from: <https://www.phe.gov/about/amcg/BARDA-BAA/Pages/barda-baa-process.aspx>.

[5] Global Biodefense. PRISM: Advancing Nucleic Acid Vaccine Platform Technologies. 2017. [Cited 2019 16 December]. Available from: <https://globalbiodefense.com/2017/11/27/prism-advancing-nucleic-acid-vaccine-platform-technologies/>.

[6] DARPA. Pandemic Prevention Platform (P3). 2017. [Cited 2019 16 December]. Available from: <https://www.darpa.mil/program/pandemic-prevention-platform>.

[7] Saul A, O’Brien KL. Prioritizing vaccines for developing world diseases. Vaccine 2017;35(1):A16-A19. <https://doi.org/10.1016/j.vaccine.2016.10.087>.

[8] Hume, H.K.C., & Lua, L.H.L. (2017). Platform technologies for modern vaccine manufacturing. Vaccine 2017;35(35 Pt A):4480-4485. <https://doi.org/10.1016/j.vaccine.2017.02.069>.

[9] Marston HD, Folkers GK, Morens DM, Fauci AS. Emerging viral diseases: confronting threats with new technologies. Sci. Transl. 2014;6(253):253ps10. [DOI:10.1126/scitranslmed.3009872](https://stm.sciencemag.org/content/6/253/253ps10).

[10] Plotkin S, Robinson JM, Cunningham G, Iqbal R, Larsen S. The complexity and cost of vaccine manufacturing—an overview. Vaccine 2017;35:4064–4071. <https://doi.org/10.1016/j.vaccine.2017.06.003>.

[11] Gilbert SC, Warimwe GM. Rapid development of vaccines against emerging pathogens: The replication-deficient simian adenovirus platform technology. Vaccine 2017;35(35 Pt A):4461-4464. <https://doi.org/10.1016/j.vaccine.2017.04.085>

[12] Von Gabain A, Klade C. Development of novel vaccines. Skills, knowledge and translational technologies. New York: Springer; 2012.

[13] Cicchetti DV. Guidelines, criteria, and rules of thumb for evaluating normed and standardized assessment instruments in psychology. Psychol Assess 1994;6(4):284-290. <https://doi.org/10.1037/1040-3590.6.4.284>.

[14] Mangham LJ, Hanson K, McPake B. How to do (or not to do) … Designing a discrete choice experiment for application in a low-income country. Health Policy Plann 2009;24(2):151–158. <https://doi.org/10.1093/heapol/czn047>.

[15] Carlsson F, Martinsson P. Design techniques for stated preference methods in health economics. Health Econ 2003;12:281-94. <https://doi.org/10.1002/hec.729>.

[16] Burgess L, Street DJ. (2005). Optimal designs for choice experiments with asymmetric attributes. J Stat Plan Inference 2005;134:288-301. <https://doi.org/10.1016/j.jspi.2004.03.021>.

[17] Street DJ, Burgess A, Louviere JJ. (2005). Quick and easy choice sets: Constructing optimal and nearly optimal stated choice experiments. Inte J Res Mark 2005;22: 459-70. <https://doi.org/10.1016/j.ijresmar.2005.09.003>.

[18] Tervonen T, Schmidt-Ott T, Marsh K, Bridges JFP, Quaife M, Janssen E. Assessing Rationality in Discrete Choice Experiments in Health: An Investigation into the Use of Dominance Tests. Value in Health 2018;21(10):1192–1197. <https://doi.org/10.1016/j.jval.2018.04.1822>.

[19] Ringuest JL, Graves SB, Case RH. Mean–Gini analysis in R&D portfolio selection. Eur J Oper Res 2004;154:157–169. <https://doi.org/10.1016/S0377-2217(02)00708-7>.

[20] Shalit H, Yitzhaki S. Evaluating the mean–Gini approach to portfolio selection. Int J Financ 1989;1(2):16-31.
